# Study of the Adhesion of the Human Gut Microbiota on Electrospun Structures

**DOI:** 10.3390/bioengineering9030096

**Published:** 2022-02-26

**Authors:** Francesco Biagini, Marco Calvigioni, Carmelo De Maria, Chiara Magliaro, Francesca Montemurro, Diletta Mazzantini, Francesco Celandroni, Monica Mattioli-Belmonte, Emilia Ghelardi, Giovanni Vozzi

**Affiliations:** 1 Research Center “E. Piaggio”, University of Pisa, Largo Lucio Lazzarino 1, 55122 Pisa, Italy; francesco.biagini@phd.unipi.it (F.B.); carmelo.demaria@unipi.it (C.D.M.); chiara.magliaro@centropiaggio.unipi.it (C.M.); francesca.montemurro@unipi.it (F.M.); 2 Department of Information Engineering, University of Pisa, Via G. Caruso 16, 56122 Pisa, Italy; 3 Department of Translational Research and New Technologies in Medicine and Surgery, University of Pisa, Via San Zeno 37, 56127 Pisa, Italy; marco.calvigioni@med.unipi.it (M.C.); diletta.mazzantini@med.unipi.it (D.M.); francesco.celandroni@dps.unipi.it (F.C.); emilia.ghelardi@med.unipi.it (E.G.); 4 Department of Clinical and Molecular Science—DISCLIMO Università Politecnica delle Marche, Via Tronto 10/A, 60126 Ancona, Italy; m.mattioli@staff.univpm.it

**Keywords:** gut microbiota, electrospinning, biofabrication

## Abstract

Although the adhesion of bacteria on surfaces is a widely studied process, to date, most of the works focus on a single species of microorganisms and are aimed at evaluating the antimicrobial properties of biomaterials. Here, we describe how a complex microbial community, i.e., the human gut microbiota, adheres to a surface to form stable biofilms. Two electrospun structures made of natural, i.e., gelatin, and synthetic, i.e., polycaprolactone, polymers were used to study their ability to both promote the adhesion of the human gut microbiota and support microbial growth in vitro. Due to the different wettabilities of the two surfaces, a mucin coating was also added to the structures to decouple the effect of bulk and surface properties on microbial adhesion. The developed biofilm was quantified and monitored using live/dead imaging and scanning electron microscopy. The results indicated that the electrospun gelatin structure without the mucin coating was the optimal choice for developing a 3D in vitro model of the human gut microbiota.

## 1. Introduction

Biofilms are complex microbial communities formed by the cooperation of microorganisms growing on a surface [1]. Their formation is typically exploited as an index of culture well-being [2]. In addition, in humans, biofilms are the major cause of microbial infections (e.g., endocarditis, kidney infections, and prosthesis-related infections) [3]. Typically, microorganisms arranged in a stable biofilm are resistant to the host immune system and are refractory to drug eradication; thus, antibiotic-resistant strains could be selected during the treatment with high antibiotic doses [4]. The bacterial adhesion process on surfaces is related to a combination of physical (e.g., van der Waals interactions, electrostatic forces) and chemical (e.g., adhesive characteristics proper of bacteria) forces, which are strengthened by the presence of extracellular matrices and typical appendages on the bacterial cells (i.e., fimbriae, pili, and flagella) [5,6]. Thus, the environment and the characteristics of the substrate may play a crucial role in the first phases of attachment. In particular, the physio-chemical properties of the material constituting the substrate (e.g., surface charge, wettability, stiffness, roughness), as well as the presence of topological features on its surface (e.g., nanopillars, honeycomb structures, squares), can promote or inhibit microbial adhesion [7,8,9,10,11,12,13,14,15,16]. As an example, Yuan et al. [10] cultured *Escherichia coli* on polystyrene samples with different wettabilities and showed that super-hydrophilic and super-hydrophobic materials caused poor bacterial adhesion compared to a moderate hydrophobicity. Yang et al. [16] tested *E. coli* and *Staphylococcus aureus* growth and biofilm formation on a honeycomb structure fabricated through a photolithography process on silicon, thus demonstrating that micropatterns with features less than 1 µm reduced bacterial adhesion and proliferation.

Among the fabrication techniques available, electrospinning is used in tissue engineering to produce fibrous scaffolds from a wide range of polymers [17]. The main advantages in exploiting the electrospinning process for improving biofilm formation rely on the production of micro- and nanostructures with a large surface area [18]. Indeed, different works suggested that the high aspect ratio with a random microporosity characterizing the structures obtained through electrospinning may furnish an effective artificial environmental niche for bacterial growth and proliferation. De Cesare et al. studied the adhesion of *Burkholderia terricola* on poly(ε-caprolactone) nanofiber mats [19]. In previous work, we demonstrated that gelatin electrospun structures are able to promote the adhesion and proliferation of the microorganisms constituting the human gut microbiota [2].

Regarding the chemical features affecting the bacterial adhesion on a substrate, different molecules were tested for their influence on different bacteria strains [20,21,22]. In particular, mucins, which are large glycoproteins contained in the human mucus, were widely studied [23,24]. Boekhorst et al. [25] demonstrated the ability of *Lactobacillus reuteri* to bind to mucins in vitro for the presence of mucus-binding domains on the outer membrane. 

However, the majority of the works reported in the literature evaluate the adhesion and proliferation of a single bacterial strain on electrospun structures, thus neglecting any effects due to the interaction of the diverse microbial communities constituting the biofilm. 

To this end, this work aimed at assessing the adhesion of human gut microbiota microorganisms on substrates that differ in both their chemical and their physical and mechanical properties. As such, we tested natural (i.e., gelatin [2]) and synthetic (i.e., polycaprolactone (PCL)) polymers to fabricate two different electrospun structures. Both of them were coated with a solution of mucins to change the chemical features of their surface, with the aim to identify the optimal environment for developing a three-dimensional in vitro model of the human gut microbiota. To assess the adhesion and microbial proliferation on the electrospun structures, crystal violet biofilm assays and imaging acquisitions (i.e., live/dead imaging and scanning electron microscopy (SEM)) were carried out.

## 2. Materials and Methods

### 2.1. Electrospun Structures Fabrication

Gelatin and PCL were used to fabricate the scaffolds. They were chosen because of their capability of being electrospun and to test the behavior of the microorganisms on supports with different physical/chemical natures [26,27]. The electrospinning process was chosen to recreate a microstructure where the bacteria could colonize thanks also to the great surface area that enhanced the adhesion sites. 

Gelatin structures were produced following the protocol proposed by Biagini et al. [2]. Briefly, gelatin scaffolds were produced by electrospinning a 10% *w*/*v* solution of gelatin (type A from porcine skin, Sigma-Aldrich, Milan, Italy) in 9:1 *v*/*v* glacial acetic acid (99.8%, Sigma-Aldrich, Milan, Italy) and demineralized water. Then, 3.68% *v*/*v* GPTMS ((3-glycidoxypropyl)-trimethoxysilane) (97%, Alfa Aesar, Kandel, Germany) was added after complete dissolution as a gelatin crosslinker and mixed at room temperature (RT) for 40 min. The gelatin-based solution was electrospun using an electrospinning apparatus (Linari Engineering, Livorno, Italy) for 1 h under an applied DC voltage of 30 kV and a feeding rate of 1.2 mL/h and a distance of 10 cm between the metal spinneret (21-gauge needle) and the collector, which was made of an aluminum foil. 

PCL structures were produced by electrospinning a 23% *w*/*v* solution of PCL (Sigma-Aldrich, Milan, Italy) in glacial acetic acid (99.8%, Sigma-Aldrich, Milan, Italy) [28]. After complete dissolution, the electrospinning process was set for 1 h under an applied DC voltage of 45 kV and a feeding rate of 0.6 mL/h. A distance of 20 cm between the metal spinneret and the collector was used. In both cases, electrospun structures were left to dry over a week at RT to reach complete solvent evaporation.

To tune the chemical surface properties of both the gelatin and PCL electrospun structures, a 5% mucin (Sigma-Aldrich, Milan, Italy) solution in deionized water was first autoclaved and then cast on half of the sterilized (see next section) electrospun structures. 

### 2.2. Contact Angle Measurement and Mechanical/Physical Characterization

To define the wettability of the structures’ surfaces, the contact angle was evaluated using a Theta Lite tensiometer (Biolin Scientific, Gothenburg, Sweden). The gelatin and PCL electrospun structures in a dry condition were cropped and tested using an RPMI 1640 culture medium (Sigma-Aldrich, Milan, Italy). The same structures, coated with the mucin solution, were also tested.

Mechanical characterization of the gelatin and PCL structures was carried out following the same testing protocol reported in [2]. Briefly, tensile tests were performed on samples in a dry condition with width × gauge lengths of 1 cm × 8 cm by using a Zwick/Roell mod Z005 equipped with a 100 N load cell. The mean thickness of each structure was evaluated using a micrometer with a precision of 10 μm. An initial grip-to-grip separation of about 4 cm was used and the crosshead speed was set to have a deformation rate of 0.1 min^−1^, calculated relative to the initial length of the sample. For each specimen, a stress–strain curve was obtained and the elastic modulus was derived as the slope of the first linear part of the curve. 

Fiber diameters and porosity of the gelatine and PCL electrospun structures were also evaluated from SEM images using ImageJ software (NIH, Stapleton, NY, USA). In particular, the porosity was calculated as the ratio between the volume of empty spaces (i.e., no fibers) and the total volume.

### 2.3. Microbiota Preparation from Stool Samples

Potential stool donors (<60 years) underwent a medical interview to exclude any history of gastrointestinal, neurological, or metabolic disorders, as well as any associated risk factors. For each donor, a blood sample and a stool sample were screened for infectious diseases 4 weeks before donation, according to the recent European Guidelines for fecal microbiota transplantation [29]. No recent exposure (<3 months) to antibiotics, immunosuppressants, or chemotherapy was also mandatory. Considering these exclusion criteria, a single healthy donor was selected. Fecal samples were collected and processed anaerobically within 6 h after defecation [29]. Briefly, 30 g of fresh feces were dissolved in 150 mL of 0.9% *w*/*v* NaCl, filtered with sterile gauzes to remove the larger corpuscular particles, analyzed to ensure the absence of pathogenic microorganisms using multiplex PCR amplification (FilmArray GI Panel, Biomérieux, France), and stored at −80 °C in 10% *v*/*v* glycerol. 

### 2.4. Microbial Growth on Electrospun Structures

Both gelatin and PCL electrospun structures were cropped to fit in a 24-well plate (Corning, NY, USA). The structures were sterilized using 2 mL of 70% *v*/*v* ethanol (Sigma-Aldrich, Milan, Italy) solution and incubated for 15 min in a sterile environment. Ethanol was removed and the wells were exposed to UV light for a further 15 min in a sterile environment. Some gelatin and PCL electrospun structures were coated with a sterile solution of 5% *w*/*v* of mucins and incubated for 16 h at 4 °C for mucin immobilization on the scaffolds. After incubation, the mucin solution was removed and wells were washed with 1 mL of sterile PBS. Then, 1.9 mL of RPMI 1640 culture medium (Sigma-Aldrich, Milan, Italy) was added to each well. Then, 100 µL aliquots of fecal microbiota suspensions were inoculated in the multi-well plates prepared as described above. Sterility controls made of microorganism-free electrospun structures and 2 mL of medium were also included. Plates were incubated at 37 °C in an anaerobic atmosphere by using Oxoid AnaeroGen (Thermo Fisher Scientific, Waltham, MA, USA) for a total of 7 days. Every 72 h, plates were opened and 670 µL of the medium were replaced with an equal volume of fresh medium. 

### 2.5. Biofilm Biomass Measurement

The evaluation of microbial adhesion to the electrospun structures with and without mucins was assessed using a crystal violet assay at different time points (24 h, 48 h, 72 h, and 7 days) [30]. The culture medium was first removed, and then non-adherent planktonic microorganisms were eliminated by washing the wells three times with 1 mL of PBS. Microbial biofilms on the electrospun structures were stained using 2 mL of 0.1% *w*/*v* crystal violet (Carlo Erba, Milan, Italy) for 30 min at RT [30]. Wells were washed three times with 1 mL of deionized water and covered with 2 mL of absolute ethanol for 15 min at RT to solubilize the crystal violet. Aliquots of ethanol–crystal violet solution (200 μL) were transferred to a 96-well plate and the optical density at 570 nm (OD_570_) was measured using a microplate reader (Multiskan FC, Thermo Fisher Scientific, Waltham, MA, USA) [31,32]. The absorbance was adjusted by subtracting the mean OD_570_ of the sterile controls to the OD_570_ from each sample. 

### 2.6. Live/Dead and Scanning Electron Microscopy Imaging

For live/dead imaging, a Cell MeterTM Bacterial Viability Assay Kit (AAT Bioquest, Sunnyvale, CA, USA) was used. Samples, taken at different time points (24 h, 48 h, 72 h, and 7 days), were prepared following the product’s protocol. Briefly, after supernatant removal, wells were washed three times with 1 mL of TBS and the working solution was cast on each well and incubated at RT for 30 minutes while protected from light. Images were acquired by using a Nikon A1 confocal microscope (Nikon, Tokyo, Japan) equipped with a 10× objective (pixel size: 1.24 µm). Viable bacteria emit green fluorescence (Ex/Em 510/530) due to the *MycoLight* Green fluorophore, while the dead ones, whose cellular membranes are damaged, incorporate propidium iodide and emit red fluorescence (Ex/Em 600/660). 

For the SEM imaging, wells were first washed three times with 1 mL of PBS. Microorganisms were then fixed by adding 1 mL of 2% *w*/*v* paraformaldehyde (PFA, Sigma-Aldrich, Milan, Italy) and incubating at 4 °C for 16 h in a dark room. After the PFA removal, wells were washed three times with 1 mL of PBS. Fixed samples were covered with a solution of 2.5% *v*/*v* glutaraldehyde (Sigma-Aldrich, Milan, Italy) in PBS, post-fixed in 1% *w*/*v* osmium tetroxide (Sigma-Aldrich), and dehydrated in increasing ethanol concentrations and hexamethyldisilazane (HMDS) (Sigma-Aldrich, Milan, Italy). For the acquisition, samples were mounted on aluminum stubs, gold sputtered using the Edwards Sputter Coater B150S equipment, and observed with a Philips XL 20 SEM microscope (FEI Italia SRL, Milan, Italy) at 2000× magnification. Images from negative controls (i.e., scaffolds without bacteria) were also acquired.

Both images from confocal microscopy and SEM were analyzed using ImageJ software (NIH, Stapleton, NY, USA).

### 2.7. Statistical Analysis

Experiments were carried out in triplicate and data are expressed as the mean ± standard deviation. Statistical analysis was performed with GraphPad Prism 8 (GraphPad Software Inc., San Diego, CA, USA). For experiments related to biofilm formation, one-way analysis of variance (ANOVA) followed by the Tukey–Kramer post hoc test was performed. Statistical significance was set at a *p*-value of <0.05.

## 3. Results

The physical and mechanical characterization of electrospun scaffolds is reported in Table 1.

**Figure 1:**
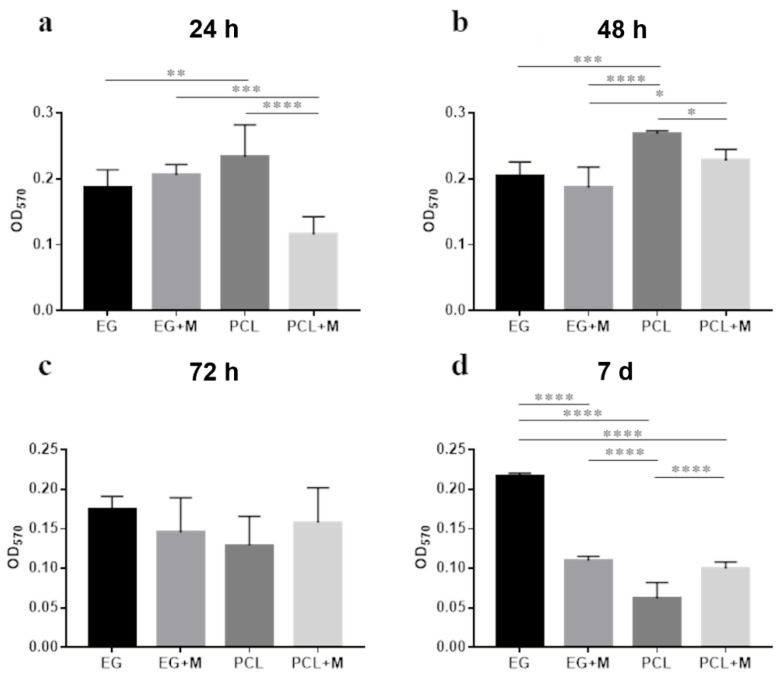
Analysis of the microbial biofilm formation using crystal violet quantification of the fecal microbiota at (**a**) 24 h, (**b**) 48 h, (**c**) 72 h, and (**d**) 7 days of incubation on different structures (electrospun gelatin structures, EG; electrospun PCL structures, PCL; electrospun gelatin structures with mucins, EG + M; electrospun PCL structures with mucins, PCL + M). (* *p* < 0.05, ** *p* < 0.01, *** *p* < 0.001, **** *p* < 0.0001).

The crystal violet assay for the quantification of microbial biofilms was used to evaluate the microbial ability to form multi-layered consortia on both gelatin and PCL structures with and without mucins. Figure 1 shows the quantification of the fecal microbiota biomass adhered to the electrospun structures at different time points. At 24 h and 48 h (Figure 1a,b, respectively), the biofilm biomass was significantly more abundant on the electrospun PCL structures compared to the other scaffolds. However, no significant difference was recorded between the samples at 72 h of incubation (Figure 1c), and the gelatin structures without mucins were found to be significantly more suitable for biofilm growth than the other materials after 7 days of incubation (*p* < 0.0001, Figure 1d). The presence of mucins on both gelatin and PCL seemed to negatively affect the formation of a biofilm from the fecal microbiota. Mucins on the gelatin membranes contributed to reducing the adhesion to supports than gelatin alone at 7 days (*p* < 0.0001). On PCL substrates, mucins produced a significant reduction in microbial adhesion compared with PCL alone at both 24 h (*p* < 0.0001) and 48 h (*p* < 0.05). These data indicate that electrospun gelatin structures in the absence of a mucin coating are more suitable than the other structures used in this work in supporting the adhesion and growth of the fecal microbiota for prolonged times of in vitro culture, thus suggesting their supportive role, especially in the later stages of biofilm formation (A complementary representation of the same data, grouping together all the scaffold analyzed at same time points is reported in Figure S1).

**Figure 2:**
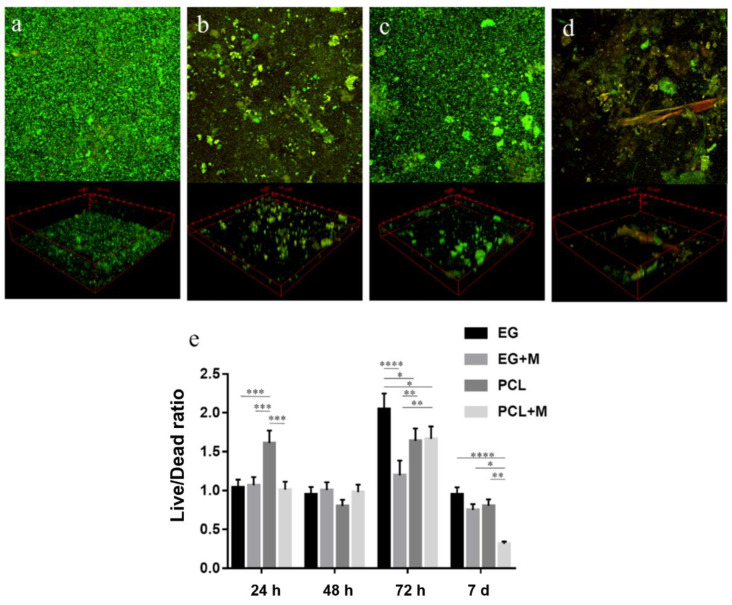
Live (green)/dead (red) images of the fecal microbiota cultured on the electrospun structures at 7 days (z-stack images and 3D reconstruction). (**a**) Electrospun gelatin structure; (**b**) electrospun gelatin structure with mucin; (**c**) electrospun PCL structure; (**d**) electrospun PCL structure with mucin; (**e**) ratio between live and dead microorganisms at different time points. (* *p* < 0.05, ** *p* < 0.01, *** *p* < 0.001, **** *p* < 0.0001).

Regarding the live/dead images, the viability of the microbial consortia on the electrospun structures was evaluated by calculating the ratio between the number of green and red objects, corresponding to live and dead microbes, respectively, on the entire 3D stacks. The objects were identified in the green and red channels with the ImageJ 3D segmentation plugin. Figure 2 shows the z-stack images of the different structures at 7 days and the corresponding live/dead ratio (images of other time points are provided in the Supplementary Information). All the scaffolds investigated seem to be suitable for recreating a three-dimensional environment that allowed for the formation of a multi-layered biofilm persisting for at least 7 days (Figure 2a–d). Indeed, the ratio was higher for all structures at 72 h compared to 7 days (Figure 2e) and live bacteria were more numerous than dead bacteria, i.e., a ratio over one, at all the time points, except for day 7 and the electrospun PCL structure at 48 h (For further details regarding the other time points see Figures S2–S4). 

**Figure 3:**
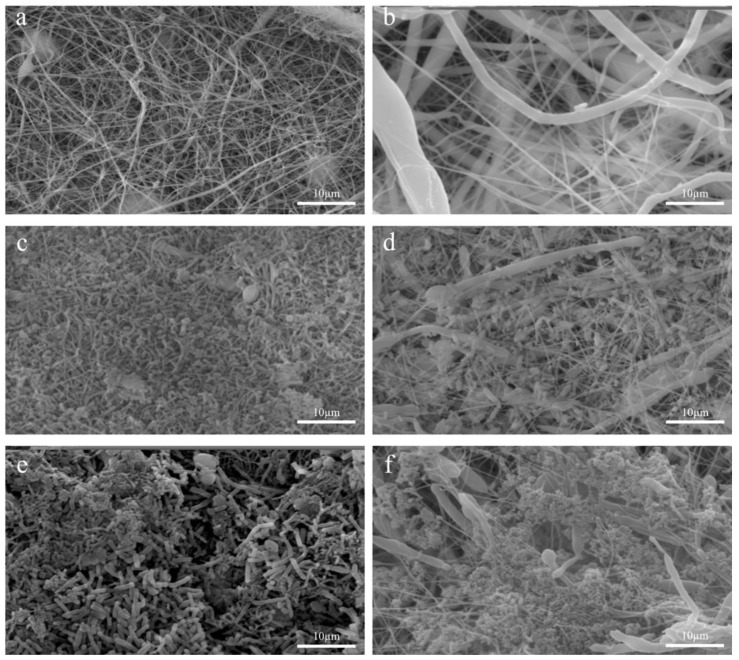
SEM images of the fecal microbiota cultured on the electrospun structures at 7 days. (**a**) Electrospun gelatin structure without bacteria; (**b**) electrospun PCL structure without bacteria; (**c**) electrospun gelatin structure; (**d**) electrospun PCL structure; (**e**) electrospun gelatin structure with mucins; (**f**) electrospun PCL structure with mucins.

SEM observations highlighted morphological and quantitative differences in the bacterial population. In particular, Figure 3a,b show the SEM images of gelatin and PCL structures without bacteria and mucins. Figure 3c–f show the in-vitro-cultured fecal microbiota on the different structures at 7 days (images of other time points are provided in the Supplementary Materials). No alterations were observed between samples at 24 h and 48 h. However, increased bacterial colonization was found in all structures at 72 h and 7 days (For further details regarding the other time points see Figures S5–S7). 

## 4. Discussion

In this work, the adhesive ability of the human gut microbiota on electrospun substrates with different chemical and mechanical properties with and without the addition of a glycoprotein (i.e., mucin) was tested. Typically, bacterial adhesion on a substrate is assessed to define whether a biocompatible surface has antimicrobial properties, especially for implantable biomaterials or cell scaffold applications [33,34]. Here, our goal was to identify the optimal substrate for bacterial adhesion to recreate an in vitro model of the human gut microbiota. However, these findings could find applications in other studies, ranging from fermentative experiments, pharmacological testing, and crosstalk interactions between inhabiting microorganisms and the host [35,36]. To reach this goal, we used electrospun structures, which are well known to improve bacterial adhesion due to their micro and nano random structures and a high surface/volume ratio [2,19,37].

Two materials with different natures (i.e., gelatin, which is a natural protein, and PCL, which is a synthetic polymer) were used to test and compare how the microorganisms reacted to different surfaces. The mechanical behavior was found to be significantly different between the gelatin and PCL structures in terms of the elastic modulus. In addition, the contact angle test showed that the gelatine structures were hydrophilic, while the PCL structures were hydrophobic. Although data from the literature are in contrast to the use of materials with high/low elastic modulus for bacterial culture [7,11,38,39], hydrophobic materials are typically indicated as good substrates for culturing microorganisms [10,40,41]. However, these observations usually refer to a bacterial monoculture, while our data were taken from a complex multispecies culture [42]. 

The biofilm formation on the PCL structures was higher than the gelatin ones at 24 and 48 h, while after 7 days of incubation, the adhered biomass was significantly more abundant on the gelatin-made scaffolds. While adhesion on the PCL structures was higher at 24 h and 48 h compared to the other time points, the gelatin structures maintained a constant biofilm at all the time points (Figure S1 in Supplementary Materials). To the best of our knowledge, such behavior has never been described in the literature. We suggest that it can be related not only to the wettability properties of the structure but also to the chemical nature of the material itself, i.e., natural protein vs. synthetic polymer. Changes in biofilm formation on the electrospun structures between 48 h and 7 d may also be a consequence of the long-term culturing, which differs from most of the works on microbial adhesion available to date. 

Mucins were also tested as a coating for the gelatin and PCL electrospun structures to chemically modify the attachment of the microorganisms to the surface. Different works already showed the role of mucins in the adhesion of some microorganisms to a surface [23,24,25]. The mucin coating enhanced the adhesion of the human gut microbiota at the first two time points, but the adhered biomass was strongly decreased at 72 h and 7 days on both types of structure (Figure S1 in Supplementary Materials). Here, we underline that the mucins are not always associated with a more stable and persistent microbial adhesion on a surface [24,43], suggesting that the adhesive properties of mucins are not sufficient to improve the adhesion of a complex microbial population, such as the human gut microbiota, for a long culture period, but other factors are probably involved.

The results on biofilm formation were coupled to those obtained through imaging. In particular, in the live/dead confocal imaging, the formation of a multi-layered biofilm with a complex 3D structure was observed. Similar to the results from the biofilm formation assay, post-processed data from live/dead images showed that the PCL structures produced good bacterial viability in terms of bacterial survival at 24 h. In contrast, the gelatin structures were slightly better at 72 h and 7 days when compared to the other structures. The digestion of the gelatin from microorganisms, which cannot metabolize the synthetic PCL, could be the reason for this observation: once degraded from bacterial gelatinases or proteases, gelatin could be used by the human gut microbiota as a substrate for different physiological processes by increasing the microorganisms’ viability [44,45]. Overall, gelatin structures were found to be not only suitable as physical scaffolds for microbial adhesion and biofilm formation thanks to their three-dimensionality and fibrous nature, but also represent a good biocompatible material that could be exploited by bacteria as a source of nutrients.

## 5. Conclusions

In conclusion, here we present a concise study on the human gut microbiota adhesion on electrospun structures with different physical and mechanical properties. Our findings indicated that, among the electrospun structures investigated, the mucin-free gelatin structures were the most suitable for culturing human gut microbiota in terms of adhesion. This was demonstrated using a crystal violet assay on the different electrospun structures at different time points. In addition, live/dead imaging showed an increase in proliferation of the microorganisms at 72 h and 7 days on the electrospun structures, enabling the possibility to use them for long-term cultures. This work may represent a first step toward the generation of a reliable in vitro model of the human gut microbiota. In the future, this in vitro model could be exploited to obtain more complex systems, i.e., including human cells, in order to study the intricate relationship between microorganisms and the human host.

## Figures and Tables

**Table 1:** Contact angle and elastic modulus for the different electrospun structures used in the cultures. Data for the gelatin elastic modulus in dry and wet conditions are taken from [2].

**Contact Angle**	
Gelatin	28.6 ± 0.5°
PCL	109.4 ± 5.2°
Gelatin + mucin	17.5 ± 2.0°
PCL + mucin	31.7 ± 5.2°
**Elastic Modulus**	
Gelatin (dry)	23.8 ± 2.6 MPa
Gelatin (wet)	0.199 ± 0.04 MPa
PCL (dry)	2.1 ± 0.5 MPa
**Diameter of Fibers**	
Gelatin	0.32 ± 0.03 µm
PCL	0.38 ± 0.1 µm
**Porosity**	
Gelatin	31.5 ± 1.8%
PCL	23 ± 4.7%

## Data Availability

Not applicable.

## Supplementary Materials

The following supporting information can be downloaded from https://www.mdpi.com/article/10.3390/bioengineering9030096/s1, Figure S1: Analysis of the microbial biofilm formation using crystal violet quantification of the fecal microbiota for (a) EG, (b) EG + M, (c) PCL, and (d) PCL + M at different time points; Figure S2: Live (green)/dead (red) imaging of the fecal microbiota cultured on the electrospun structures at 24 h (z-stack images and 3D reconstruction); Figure S3: Live (green)/dead (red) imaging of the fecal microbiota cultured on the electrospun structures at 48 h (z-stack images and 3D reconstruction); Figure S4: Live (green)/dead (red) imaging of the fecal microbiota cultured on the electrospun structures at 72 h (z-stack images and 3D reconstruction); Figure S5: SEM imaging of the fecal microbiota cultured on the electrospun structures at 24 h; Figure S6: SEM imaging of the fecal microbiota cultured on the electrospun structures at 48 h; Figure S7: SEM imaging of the fecal microbiota cultured on the electrospun structures at 72 h.
