# Supplementary material for: Study of the Adhesion of the Human Gut Microbiota on Electrospun Structures
Source: Bioengineering (Basel). 2022 Feb 26;9(3):96. doi: 10.3390/bioengineering9030096 (PMC8945341; doi:10.3390/bioengineering9030096)
Supplement: Supplementary file 1 [file bioengineering-09-00096-s001.zip › bioengineering-1585564-supplementary.pdf]

bioengineering

MDPI

*Supplementary Materials*

# Study of the Adhesion of the Human Gut Microbiota on Electrospun Structures

**Francesco Biagini [1,2], Marco Calvigioni [3], Carmelo De Maria [1,2], Chiara Magliaro [1,2], Francesca Montemurro [1], Diletta Mazzantini [3], Francesco Celandroni [3], Monica Mattioli-Belmonte [4], Emilia Ghelardi [3] and Giovanni Vozzi [1,2,*]**

[1] Research Center "E. Piaggio" - University of Pisa, Largo Lucio Lazzarino 1, 55122, Pisa, Italy; francesco.biagini@phd.unipi.it (F.B.); carmelo.demaria@unipi.it (C.D.M.); chiara.magliaro@centropiaggio.unipi.it (C.M.); francesca.montemurro@unipi.it (F.M.)

[2] Department of Information Engineering - University of Pisa, Via G. Caruso 16, 56122, Pisa, Italy

[3] Department of Translational Research and New Technologies in Medicine and Surgery - University of Pisa, Via San Zeno 37, 56127 Pisa, Italy; marco.calvigioni@med.unipi.it (M.C.); diletta.mazzantini@med.unipi.it (D.M.); francesco.celandroni@dps.unipi.it (F.C.), emilia.ghelardi@med.unipi.it (E.G.)

[4] Department of Clinical and Molecular Science - DISCLIMO Università Politecnica delle Marche, Via Tronto 10/A, 60126, Ancona, Italy; m.mattioli@staff.univpm.it

* Correspondence: g.vozzi@ing.unipi.it

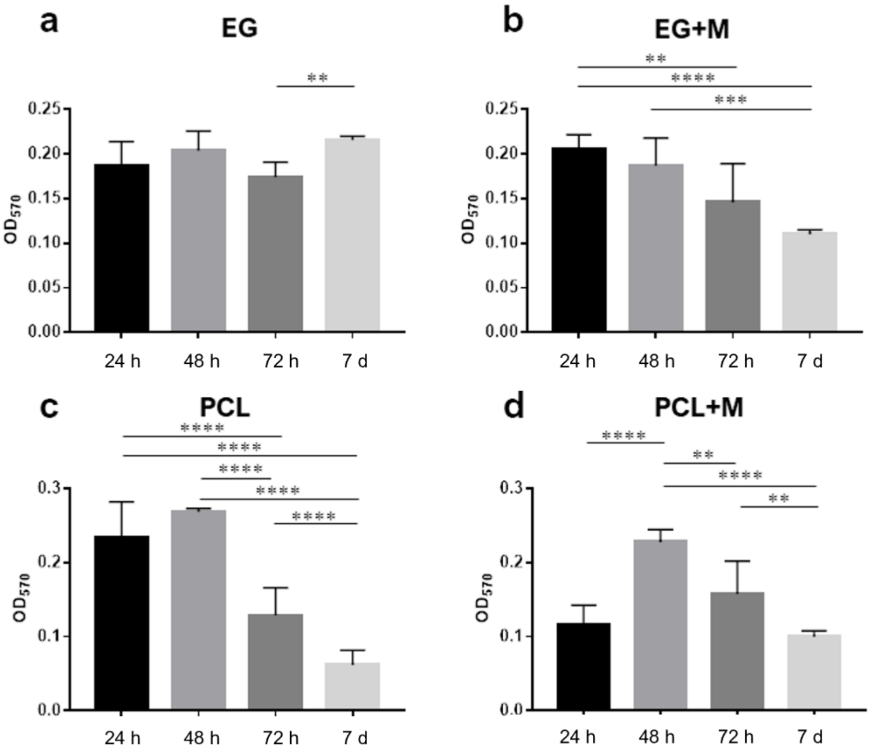


**Figure S1.** Analysis of the microbial biofilm formation using crystal violet quantification of the fecal microbiota for (**a**) EG, (**b**) EG+M, (**c**) PCL, and (**d**) PCL+M at different time points (electrospun gelatin structures, EG; electrospun PCL structures, PCL; electrospun gelatin structures with mucins, EG+M; electrospun PCL structures with mucins, PCL+M). * $p < 0.05$, ** $p < 0.01$, *** $p < 0.001$, **** $p < 0.0001$.

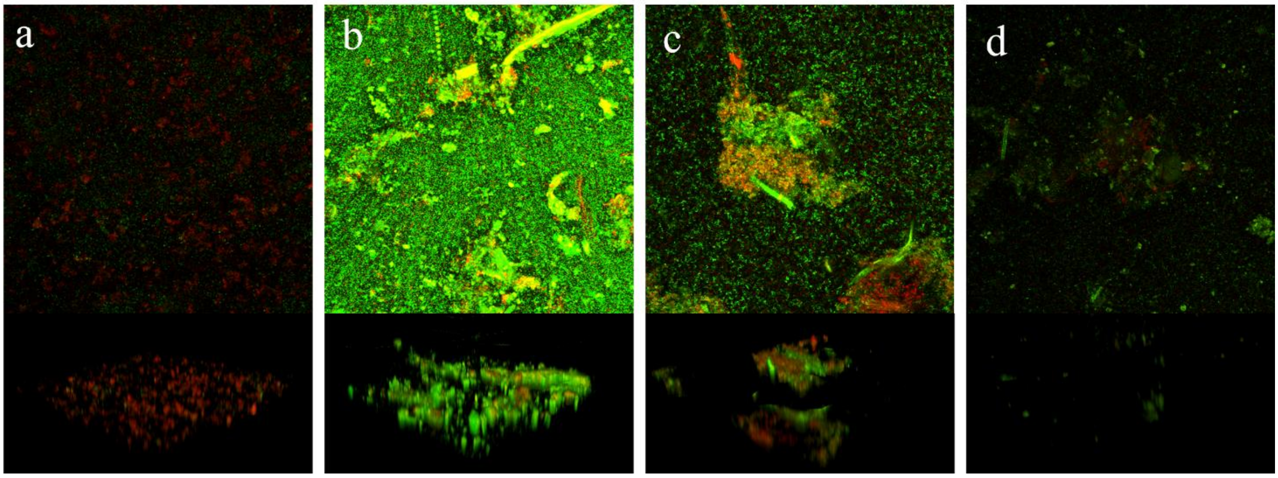


**Figure S2.** Live (green)/dead (red) imaging of the fecal microbiota cultured on the electrospun structures at 24 hours (z-stack images and 3D reconstruction). (**a**) Electrospun gelatin structure; (**b**) electrospun gelatin structure with mucin; (**c**) electrospun PCL structure; (**d**) electrospun PCL structure with mucin.

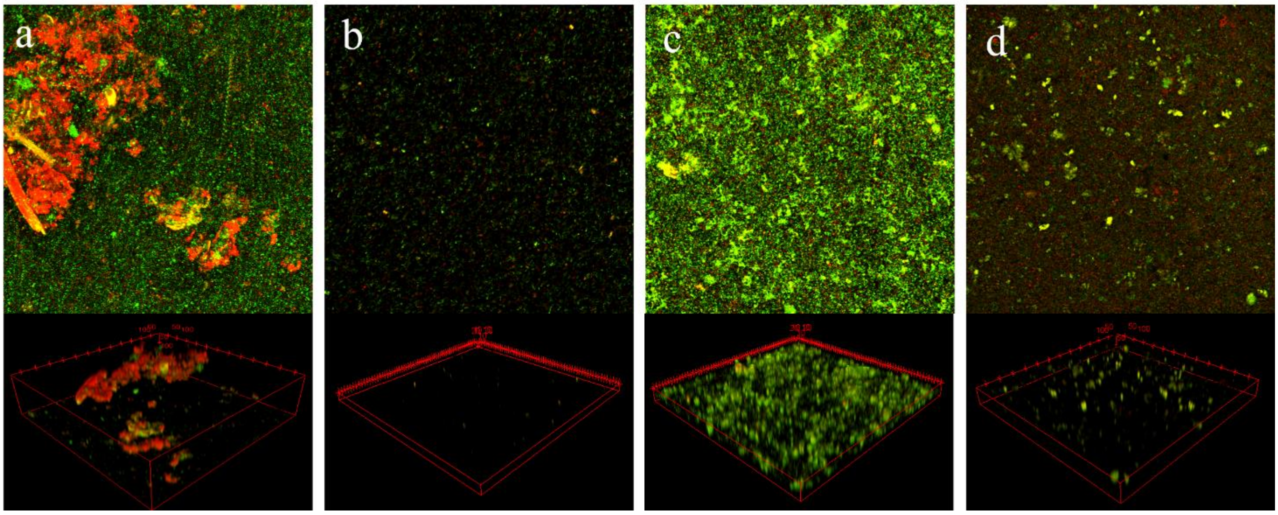


**Figure S3.** Live (green)/dead (red) imaging of the fecal microbiota cultured on the electrospun structures at 48 hours (z-stack images and 3D reconstruction). (**a**) Electrospun gelatin structure; (**b**) electrospun gelatin structure with mucin; (**c**) electrospun PCL structure; (**d**) electrospun PCL structure with mucin.

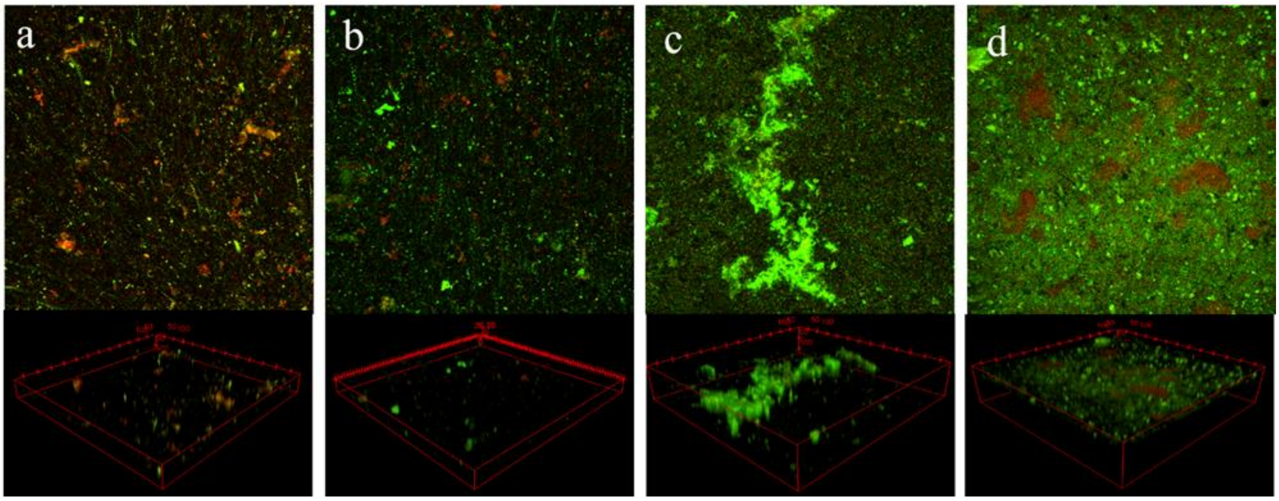


**Figure S4.** Live (green)/dead (red) imaging of the fecal microbiota cultured on the electrospun structures at 72 hours (z-stack images and 3D reconstruction). (**a**) Electrospun gelatin structure; (**b**) electrospun gelatin structure with mucin; (**c**) electrospun PCL structure; (**d**) electrospun PCL structure with mucin.

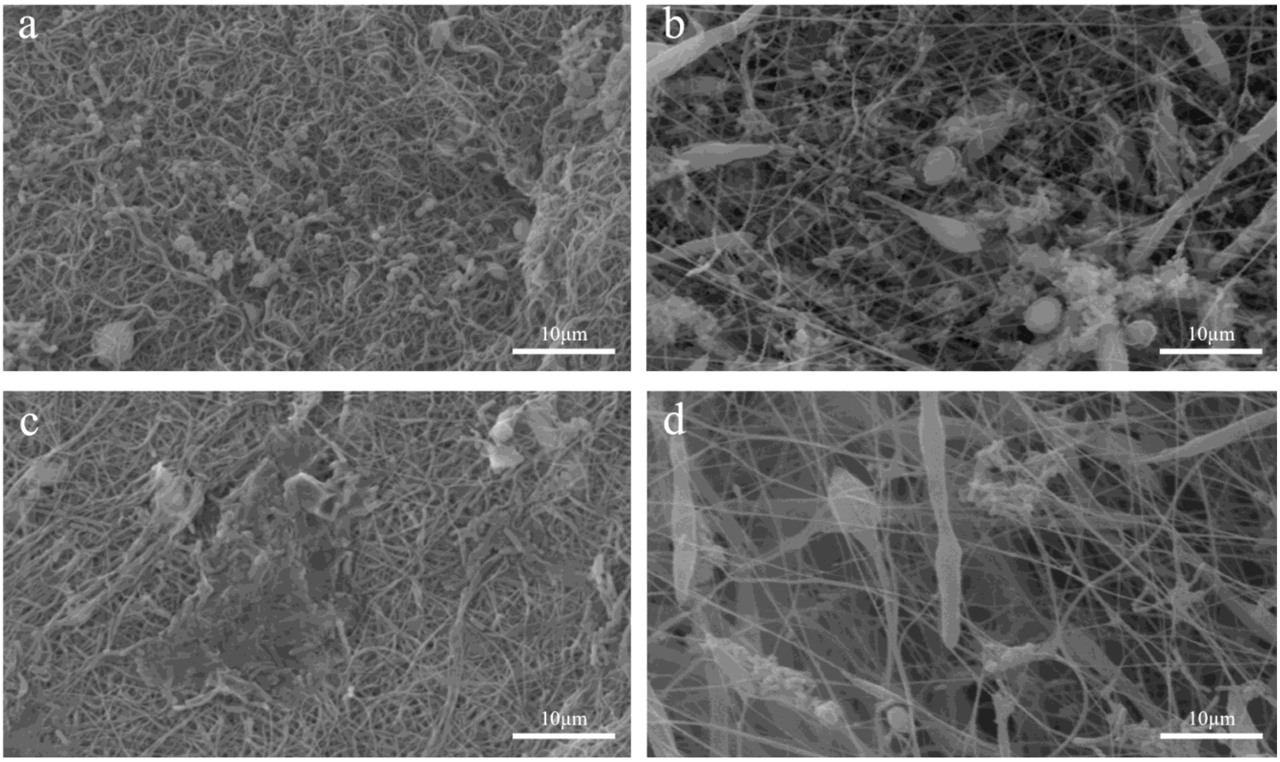


**Figure S5.** SEM imaging of the fecal microbiota cultured on the electrospun structures at 24 hours. (**a**) Electrospun gelatin structure; (**b**) electrospun PCL structure; (**c**) electrospun gelatin structure with mucins; (**d**) electrospun PCL structure with mucins.

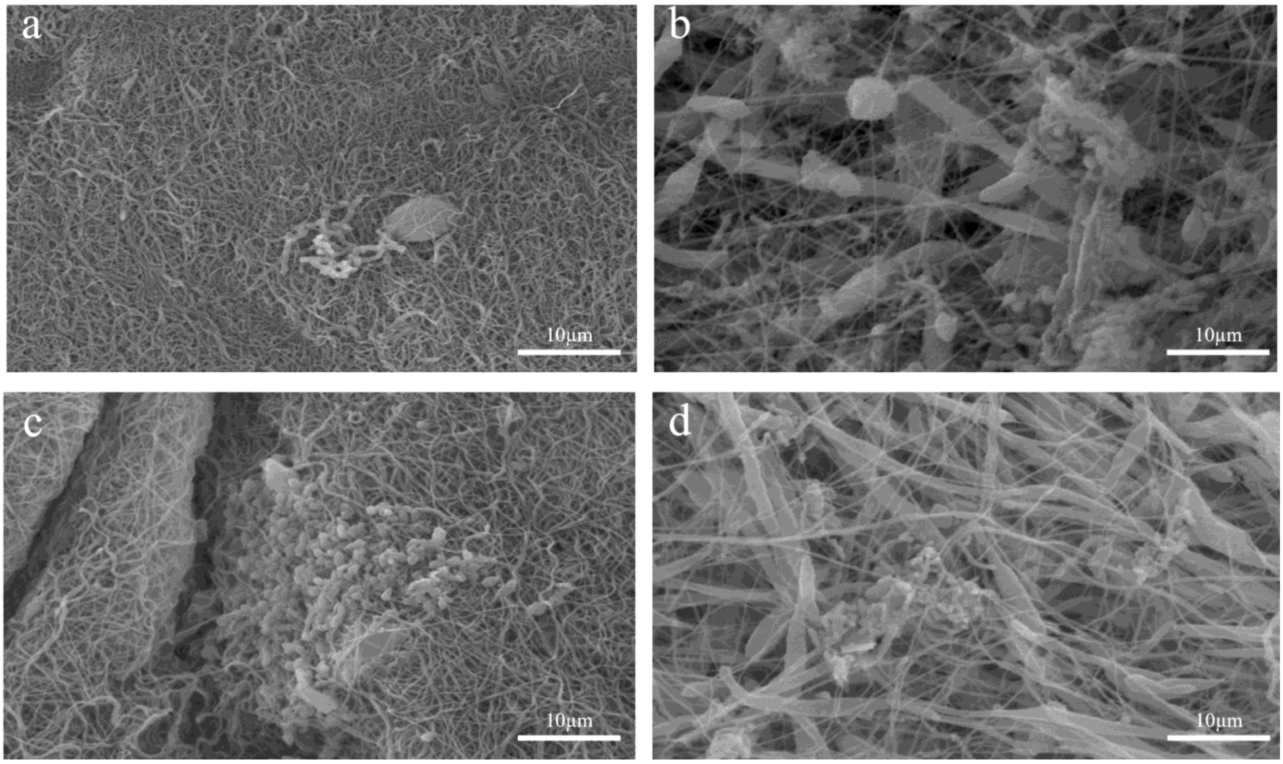


**Figure S6.** SEM imaging of the fecal microbiota cultured on the electrospun structures at 48 hours. (**a**) Electrospun gelatin structure; (**b**) electrospun PCL structure; (**c**) electrospun gelatin structure with mucins; (**d**) electrospun PCL structure with mucins.

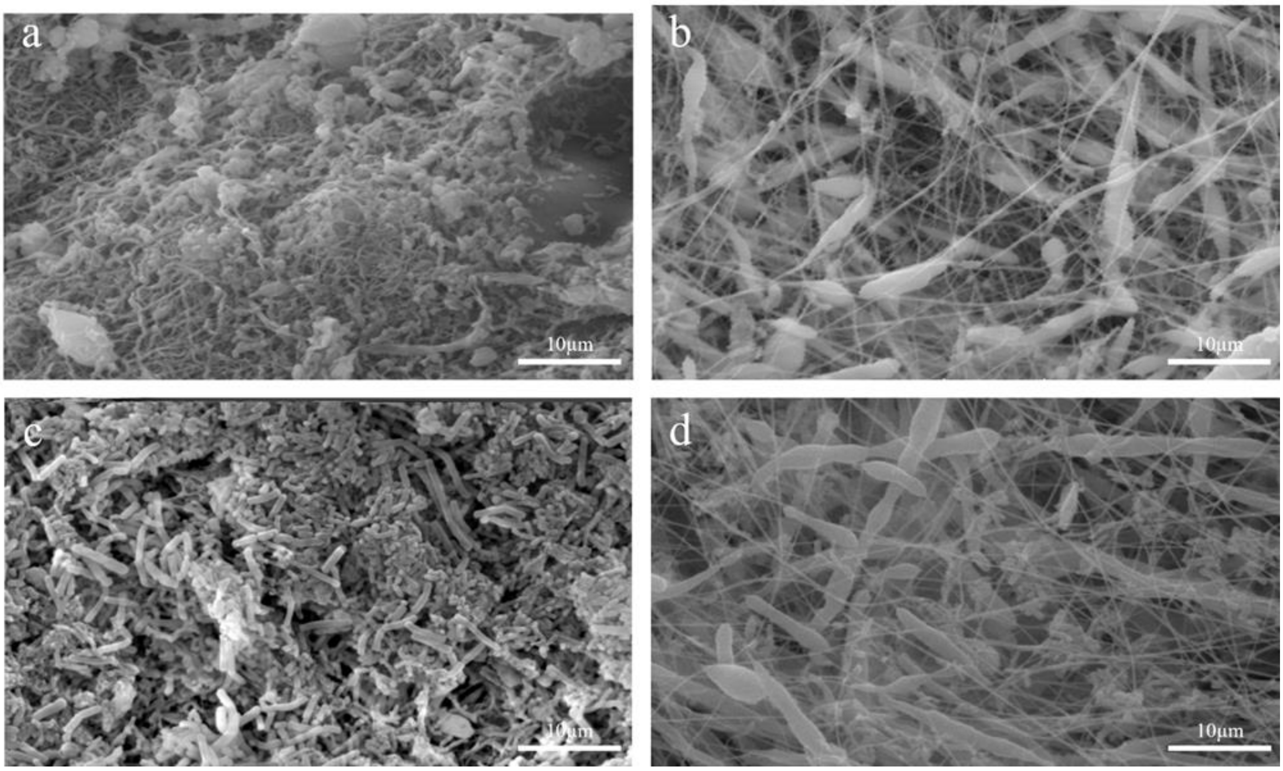


**Figure S7.** SEM imaging of the fecal microbiota cultured on the electrospun structures at 72 hours. (**a**) Electrospun gelatin structure; (**b**) electrospun PCL structure; (**c**) electrospun gelatin structure with mucins; (**d**) electrospun PCL structure with mucins.
